# Taking Medicine

**Published:** 1868-09

**Authors:** 


### TAKING MEDICINE.

Every third man or woman met on any street, on any day, will decry the use of drugs and are ready at a moment's notice to expend their wit on physic and physicians; and yet every third man or woman met on any street, on any day, will be found using drugs in some way or other; not a few intelligent persons keep by them the most deadly forms of physic to use in emergencies ; the result is that it is not an unusual newspaper announcement, "died of an overdose of medicine." This morning's paper chronicles the death of one of the most celebrated political writers in the United States, a scholar and a gentleman of influence high in office and enjoying the confidence and warm affection of a very wide circle of friends in the army and among the editorial profession. He had become habituated to the inhalation of chloroform for a painful malady, and on the present occasion suffering more than common,he took a larger amount than usual, became insensible and died in a few hours "not far from home," the home of a loving wife and six children. The counsel of this Journal from the very first number has been, never take an atom of physic on your own responsibility, but if you are sick, send at once for your family physician.

